# Towards effective digital lifestyle interventions for pregnant women with obesity: A qualitative study exploring women's and healthcare providers’ perspectives

**DOI:** 10.1177/20552076251408518

**Published:** 2026-03-23

**Authors:** Rianne J de Bruin, Caroline A Figueroa, Pam ten Broeke, Kim N de Jonge, Melek Rousian, Régine PM Steegers-Theunissen, Ageeth N Rosman

**Affiliations:** 1 Department of Obstetrics and Gynaecology, Erasmus MC, 6993 University Medical Centre, Rotterdam, the Netherlands; 2 Faculty of Technology, Policy and Management, 2860 Delft University of Technology, Delft, the Netherlands; 3 Department of Health, Medical and Neuropsychology, Faculty of Social and Behavioural Sciences, 4496 Leiden University, Leiden, the Netherlands; 4 Department of Healthcare Studies, 6985 Rotterdam University of Applied Sciences, Rotterdam, the Netherlands

**Keywords:** Pregnancy, eHealth, lifestyle care, behavioural change, co-design

## Abstract

**Background:**

Maternal obesity increases risks of adverse pregnancy outcomes and long-term diseases for mothers and child. Digital lifestyle interventions show promise, but their effectiveness depends on meeting the specific needs of pregnant women with obesity and healthcare providers (HCPs).

**Objectives:**

To explore perspectives and practices on healthy lifestyle and care for pregnant women with obesity, and to identify needs and preferences for digital lifestyle intervention development and implementation.

**Methods:**

A qualitative study using focus groups and interviews was conducted with 13 HCPs and 13 pregnant women with obesity. Sessions were audio-recorded, transcribed and analysed thematically. Women viewed a healthy lifestyle as multidimensional, encompassing nutrition, physical activity, mental well-being, and rest, but faced barriers such as pregnancy discomfort, limited knowledge, and stigma. Both women and HCPs emphasized child health as a motivator and valued goal setting and practical advice. Existing care was seen as inconsistent and generic, with HCPs constrained by time and unclear roles. Participants preferred a personalized, user-friendly mobile app with modular, evidence-based content tailored to individual goals, pregnancy stage, and medical status. Features such as self-monitoring, goal setting, and a supportive, non-judgmental tone were important. Integration into routine obstetric care was considered key for engagement and effectiveness. If designed accordingly, such tools could provide accessible, tailored support between appointments, reinforce positive behaviour change, improve patient-provider communication, and reduce HCP time pressures.

**Conclusions:**

Co-designing digital lifestyle tools with women and HCPs is vital. Personalized, feasible interventions integrated in obstetric care can support behaviour change and improve outcomes for mothers and children.

**Trial registration number:**

not applicable.

## Background

Maternal obesity is an increasing global health concern with significant consequences for both mothers and their children. It increases the risk of pregnancy and intrapartum complications, including gestational diabetes, hypertensive disorders, caesarean section and postpartum haemorrhage up to ten times. Additionally, children born to mothers with obesity are more likely to develop macrosomia and metabolic disturbances such as hypoglycaemia.^1–4^ Beyond perinatal outcomes, maternal obesity is associated with an elevated lifelong risk of non-communicable diseases, such as cardiovascular diseases and diabetes, in both mother and child.^5–7^ These risks highlight the urgent need for effective interventions to improve pregnancy outcomes and long-term health trajectories in women with obesity.

Modifying lifestyle behaviours presents a feasible and promising approach to mitigate the consequences of obesity. Though particularly challenging to address, especially during pregnancy, previous studies have demonstrated that improvements in diet, physical activity, mental well-being, and sleep can positively impact pregnancy outcomes.^8,9^ Despite this evidence, lifestyle care has not yet been fully integrated into standard obstetric care. In-person lifestyle interventions, while beneficial, require substantial time and effort from both healthcare providers (HCPs) and patients, creating barriers to widespread implementation.^10–12^

Digital health interventions, including mobile apps and telemedicine platforms, offer a promising solution for overcoming these barriers.^13–15^ These tools can provide personalized, continuous guidance, helping pregnant women effectively adopt healthier behaviours while addressing barriers such as time constraints and geographical limitations.^13,14,16,17^ Since 2011, our department has developed and evaluated *Smarter Pregnancy*, a digital lifestyle coaching program for (pre)pregnant women and their partners. Its effectiveness has been demonstrated in various populations, with improvements in nutrition, folic acid supplement use, pregnancy rates, embryonic growth, and maternal blood pressure.^16–22^ However, subgroup analyses showed lower effectiveness in women with obesity.^16,22^ This aligns with broader evidence suggesting that digital tools not tailored to the specific needs of this population may be less effective. Factors such as reduced motivation, physical discomfort, and weight-related stigma, particularly around physical activity and weight management, are commonly reported barriers among these women.^12,23,24^

To ensure effective implementation in practice, digital lifestyle interventions must be carefully tailored to meet the specific needs and preferences of women with obesity and HCPs.^25,26^ Despite this, most existing research has focused on digital interventions for pregnant women in general, without specifically addressing those with obesity.^24,27–30^ In studies that do consider this subgroup, the perspective of HCPs is often overlooked, despite their crucial role in successful implementation in clinical practice.^31,32^

To address these knowledge gaps, this study aims to (a) explore the current perspectives and practices surrounding healthy lifestyle behaviours and lifestyle care for pregnant women with obesity, and (b) explore the needs and preferences of pregnant women, former users of Smarter Pregnancy and HCPs regarding the content, functionalities, and implementation of a digital lifestyle intervention. By incorporating insights from both pregnant women with obesity and HCPs, we seek to contribute to the development of more effective, tailored, and clinically relevant digital tools that can be successfully integrated into obstetric care, ultimately aiming to prevent adverse health outcomes for mothers and children.

## Methods

### Study design

A qualitative study was conducted using focus groups and interviews. The study adhered to the standards for reporting qualitative research (SRQR)^33^ and consolidated criteria for reporting qualitative research (COREQ).

This study is part of the HYGEIA project, which aims to develop and evaluate a digital lifestyle intervention specifically tailored for pregnant women with obesity. The intervention will be tested in a randomized controlled trial (NL-OMON57196) and is based on an existing (cost-)effective lifestyle coaching program, *Smarter Pregnancy* (*Slimmer Zwanger in Dutch*),^16,17,22^ which offers women and their partners 26 weeks of personalized lifestyle support based on an initial screening of dietary habits, folic acid intake, and substance use. Participants receive tailored advice, motivational messages, practical tips, and incentives such as vouchers and seasonal recipes to encourage lifestyle improvements. As part of the HYGEIA project, Smarter Pregnancy will be extended and extensively adapted to meet the specific needs of pregnant women with obesity. The program's content, structure, and delivery will be modified according to the results presented in this paper, to improve user engagement and health outcomes, ensuring a better fit to the unique challenges faced by this population.

### Setting

A combination of focus groups and semi-structured interviews was employed to explore both collective and individual perspectives on digital lifestyle support. Three focus groups were conducted to gather a broad understanding of preferences regarding the content, functionalities, and implementation of a digital lifestyle tool. Focus groups were selected because they facilitate interactive discussions, enabling both individual opinions and shared group perspectives to emerge. The insights gained from the focus groups informed the development of a semi-structured interview guide, which allowed for more in-depth exploration of individual experiences, needs and preferences.

### Participant recruitment and selection

Participants were recruited through purposive sampling, via email, telephone, or in person and provided with study information before participation. Participants received financial compensation to cover time investment and travel expenses (50 euros for women, 25 euros for HCPs). All participants had to be proficient in Dutch to be eligible.

Participants were selected from 3 key groups.

1. Pregnant women: Women with obesity (pregestational body mass index (BMI) ≥ 30 kg/m²) who were currently pregnant. The sample aimed to represent a diverse range of pregnancy durations, BMI levels, parity, and socioeconomic backgrounds. Participants were recruited through the outpatient clinic of the department of Obstetrics and Gynaecology at Erasmus University Medical Centre and two affiliated midwifery practices in Rotterdam, the Netherlands.
2. Former users of Smarter Pregnancy: Women with obesity (pregestational BMI ≥30 kg/m²) who had used Smarter Pregnancy during a pregnancy in the previous year. These women could have been pregnant again at the time of participation. Participants were recruited through the Erasmus University Medical Centre in Rotterdam, the Netherlands.
3. Healthcare providers: HCPs involved in the obstetric care of pregnant women with obesity. Participants were recruited through the researchers’ professional networks, ensuring representation from a variety of professional backgrounds (ie obstetricians, midwives, dietitian) and regions across the Netherlands.

Both groups of women (pregnant women and former users of Smarter Pregnancy) will collectively be referred to as “women” in subsequent sections of the report, to enhance readability.

The medical ethical committee of Erasmus University Medical Centre waived the need for ethical approval after reviewing the study design (MEC-2024-0364). All participants received study information, provided written informed consent prior to participation, and were given the option to opt out of the study or withdraw their data at any time.

### Data collection

Three focus group sessions were held in July 2024. One with women, conducted in person to facilitate personal interaction. Two with HCPs, conducted online via Microsoft Teams to accommodate participants’ schedules and remove travel barriers. Sessions were moderated by one or two experienced researchers, including a medical doctor and assistant professor in digital health (CF), a psychologist (KK) and midwife and lector (AR). They were supported by two additional researchers, a medical doctor (RB) and economist (JA), who occasionally asked questions and took notes. All participants were aware of the researchers’ backgrounds and study objectives.

The focus groups lasted 90 to 120 min and followed a semi-structured topic guide. The sessions started with a general introduction. To explore current perspectives and practices surrounding healthy lifestyle behaviours and lifestyle care for pregnant women with obesity (aim a), the following topics were discussed with women: 1) the importance of health and lifestyle during pregnancy, 2) the impact of pregnancy on their health and lifestyle, 3) attempts for improvement of lifestyle, 4) experience with lifestyle care and 5) experience with pregnancy and/or lifestyle apps. Similarly, the following topics were discussed with HCPs: 1) current practices in lifestyle care, 2) barriers and facilitators in lifestyle care, 3) experiences regarding update amongst women with obesity and 4) personal opinions on the importance of lifestyle care during pregnancy.

To explore the needs and preferences regarding content, functionalities and implementation (aim b), participants first received information about Smarter Pregnancy, along with some screenshots (Additional file 1, figures 2–4). Next, they were asked about 1) their opinions on Smarter Pregnancy, 2) their experience with Smarter Pregnancy (in case of former users) and 3) their suggestions for improvement. Subsequently, they were asked about their wishes for the new intervention regarding 1) content, 2) functionalities, 3) use and 4) barriers and facilitators for implementation. To help participants organize these preferences, visual materials were used—on paper during the in-persons session or via Microsoft Whiteboard during online sessions. These materials included suggestions and examples for potential content, based on literature and existing pregnancy applications (Additional file 2). Participants were asked to categorize the examples as essential (must have), desirable (nice to have), or unnecessary, and were encouraged to contribute and classify their own ideas as well. The rationales underlying their classifications were subsequently explored in group discussions.

Twelve semi-structured interviews were conducted in person or via Microsoft Teams between August and October 2024, 7 with women and 5 with HCPs. Interviews lasted 30 to 45 min and were conducted by experienced researchers (KJ or NA for the women, RB or AR for the HCPs). A semi-structured interview guide was used, following a similar topic list as used for the focus groups (Additional files 3 and 4). Notes were taken during the interviews.

After 3 focus groups and 12 interviews, data saturation was reached, as no new themes emerged.^34^

### Data analysis

All focus groups and interviews were video- and audio recorded and transcribed verbatim by a researcher (RB) or transcription company (Amberscript.ti). Video recordings were used to accurately attribute statements to participants. Thematic analysis was conducted following the approach of Braun and Clarke.^35^ Initially, transcripts were read repeatedly for familiarization, and preliminary codes were generated using an inductive approach by one researcher (RB or KJ) and then checked by a second researcher (RB or KJ). The resulting unstructured list of initial codes was discussed with 3 other researchers (CF, AR and PB). In the second phase of analysis, codes were refined and organized into a structured coding list using both inductive and deductive approaches. Relationships between codes were identified and organized into main and subthemes by 2 researchers (RB and KJ). The resulting, structured list of candidate themes was discussed within a multidisciplinary research team (RB, CF and AR) until consensus was reached. Themes were then further refined and named, ensuring they accurately represented the complete dataset.

Atlas.TI version 25 was used for systematic data management and analysis.

## Results

### Participant characteristics

Characteristics of participants are displayed in Tables 1 and 2. In total, 13 women participated, of whom 11 were pregnant at the time of participation and 4 had used Smarter Pregnancy in a former pregnancy. Additionally, 13 HCPs from 8 different professions participated, working in 8 different institutions.

**Table 1.** Characteristics of women (n = 13)

Participant		Age (years)	Pregnancy duration or time postpartum (weeks)	Pre- pregnancy BMI (kg/m2)	Parity	**Background**	**Occupational status**
**Focus group**							
**W 1**	Pregnant	36	14	34	0	Dutch	Unemployed
**W 2**	Pregnant	29	14	33	1	Dutch	Employed
**W 3**	Pregnant	34	30	31	2	Cape Verdean	Employed
**W 4**	Pregnant	32	20	30	0	Dutch-Indian	Employed
**W 5**	Pregnant	35	9	55	0	Dutch	Employed
**W 6**	Pregnant & former user	38	19	32	1	Dutch	Unemployed
**Interviews**							
**W 7**	Former user	30	16 postpartum	34	2	Dutch	Employed
**W 8**	Former user	34	8 postpartum	30	1	Dutch	Unemployed
**W 9**	Pregnant & former user	36	21	30	0	Dutch	Unemployed
**W 10**	Pregnant	33	28	44	2	Dutch	Employed
**W 11**	Pregnant	36	34	Unknown	1	Dutch	Employed
**W 12**	Pregnant	31	30	Unknown	1	Turkish	Employed
**W 13**	Pregnant	27	28	Unknown	0	Moroccan	Unemployed

W: woman.

**Table 2.** Characteristics of HCPs (n = 13)

Participant	Specialty	**Experience (years)**	**Place of work**
**Focus group session 1**			
**HCP 1**	Obstetric lifestyle nurse practitioner	3	Academic hospital, Rotterdam
**HCP 2**	Dietitian specialized in diabetes	16	Academic hospital, Rotterdam
**HCP 3**	Diabetes specialist nurse	2	Academic hospital, Rotterdam
**HCP 4**	Diabetes specialist nurse	15	Academic hospital, Rotterdam
**Focus group session 2**			
**HCP 5**	Midwife (hospital based)	18	Non-Academic Hospital, Enschede
**HCP 6**	Midwife (hospital based)	20	Non-Academic Hospital, Rotterdam
**HCP 7**	Physiotherapist	16	General physiotherapy University of applied Sciences, Rotterdam
**HCP 8**	Gynaecologist - Perinatologist	21	Academic Hospital, Rotterdam
**Individual interviews**			
**HCP 9**	Midwife (primary care based)	21	Midwifery practice, Rotterdam
**HCP 10**	Midwife (primary care based)	26	Midwifery practice, Druten
**HCP 11**	Midwife (primary care based)	37	Midwifery practice, Tiel
**HCP 12**	Gynaecological lifestyle nurse	3	Non-Academic Hospital, Capelle a/d Ijssel
**HCP 13**	Gynaecologist	10	Non-Academic Hospital, Capelle a/d Ijssel

HCP: healthcare provider.

Four focus group participants and 1 interview participant who initially agreed to participate, ultimately did not. Two pregnant women due to pregnancy complications and 3 HCPs due to last-minute clinical obligations.

### Themes

**Figure 1.**
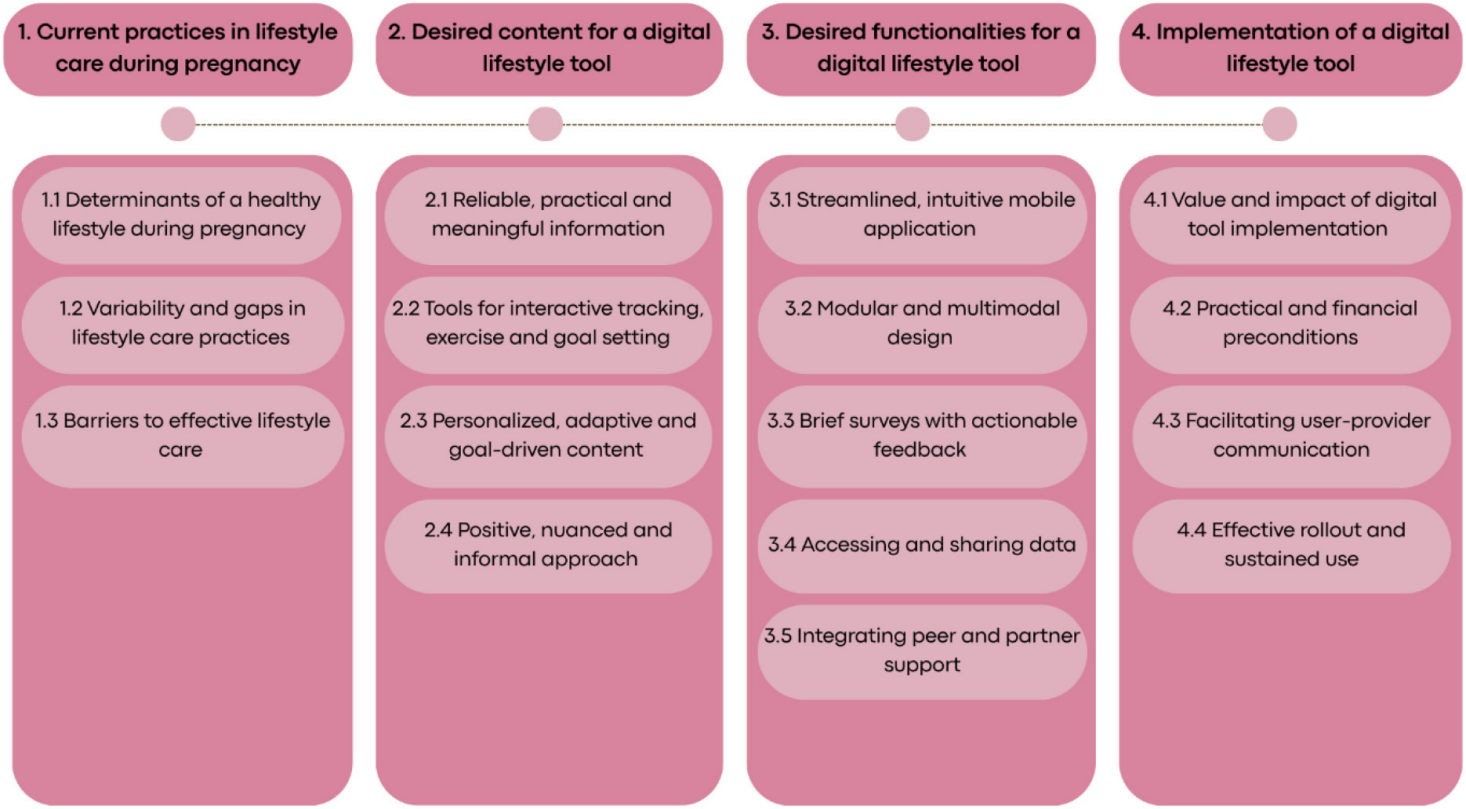
Overview of themes and subthemes.

The analysis resulted in 4 main themes and 16 subthemes, as displayed in Figure 1. These are discussed below.

#### Current practices in lifestyle care during pregnancy.

##### Determinants of a healthy lifestyle during pregnancy

Women described a healthy pregnancy lifestyle as encompassing balanced nutrition, regular physical activity, avoiding harmful substances, mental well-being, sufficient sleep, and strong social support. However, many struggled to maintain these behaviours due to pregnancy-related discomforts, such as nausea, fatigue, pelvic pain, mood swings, and stress. As W 1 put it: “*It's all connected. For example, exercise: on days when I don’t feel well, I don’t move. You couldn’t get me off the couch even with ten horses.”*

Additional barriers to healthy lifestyle identified by women and HCPs included limited knowledge about healthy choices and the risks of obesity during pregnancy, fear of judgment from others and concerns about making the wrong decisions for their baby. Economic constraints, such as the cost of healthier food or professional dietary advice, were also raised as barriers.

Despite these challenges, several strong motivators for positive behaviour change were identified. Both women and HCPs emphasized the baby's health as a central driver of behaviour change. As W 9 explained: “*It should be enough for yourself, but I think pregnant women are even more receptive when something affects their child.”* Providing more knowledge and insight, for instance on healthy weight gain, was mentioned as another motivator. Goal setting was highlighted as a powerful strategy to support engagement and motivation. W 10 shared: “*If you set a goal and achieve it, even if it seems small to others, it can be a big achievement for you. Checking something off can be beneficial for mental health, rather than always feeling like you’re not doing well enough.”* HCP 2 added: “*It helps them reflect on what goals they want to achieve and revisit them when needed. It can also serve as a source of motivation, reminding them why they started and what they want to accomplish.”*

##### Variability and gaps in lifestyle care practices

Participants reported both inconsistent integration and considerable variation in lifestyle care during prenatal care. While some HCPs routinely addressed lifestyle topics, others only discussed them when there was a clear medical indication. Some professionals admitted postponing these conversations to avoid triggering feelings of weight bias or stigma in their patients. Weight monitoring practices also varied, often for the same reasons. The type and depth of lifestyle advice provided differed considerably between providers.

Women described encounters with HCPs as predominantly problem-focused and impersonal, with limited attention to their lifestyle behaviour. W 8 noted, “*When you’re being monitored in the hospital, contact with midwives is purely medical. There's no conversation about whether you’re drinking alcohol, getting enough exercise, how you’re feeling, or if you’re experiencing pain. That kind of support was almost completely absent. It was really just about the medical aspects.”* When advice was given, women reported it was often vague and general without adaptations to individual circumstances. As W 4 illustrated: “*I understand that healthcare providers are trying to figure out how to guide us. But it still too often focuses on general and standard advice. They say: ‘You should exercise much more because you can tell you don’t just by looking…’ They don’t actually say that, but you can feel it. I cycle and walk every day. I go swimming. I don’t really know what else I should be doing. But then the conversation just ends there, and that's frustrating.”* While many women were referred to dietitians for nutrition advice, HCPs themselves acknowledged rarely offering personalized recommendations.

##### Barriers to effective lifestyle care

For HCPs, the main barriers to delivering lifestyle care were time constraints, workload, and uncertainty about their role or expertise in this area. HCP 7 reflected: “*First, I have to ask myself, is this my role? What does it bring? Do I need to let something else go? I already have so much to do and too little time*.” Some HCPs acknowledged that implicit bias and stigma towards women with obesity could affect how lifestyle advice was framed and delivered. HCP 8 noted, “*I also notice that many healthcare providers can be quite stigmatizing towards women with obesity, thinking they’re not motivated.”*

Women, in turn, expressed hesitation to discuss their challenges due to fear of judgment or punishment. They also felt that advice often failed to account for their specific needs and constraints, leading to frustration and disengagement. As W 8 shared, “*They say: ‘You should do this,’ but they don’t understand the limitations I have and how that works for me.”*

##### Desired content for a digital lifestyle tool

Participants agreed that a digital lifestyle tool for pregnant women should focus on the key lifestyle areas previously identified: nutrition, physical activity, mental well-being, sleep and substance use. Additionally, HCPs expressed a preference for including information on supplements, medications, and the impact of obesity on pregnancy to ensure comprehensive support and guidance. While the existing Smarter Pregnancy program already covers nutrition, substance use, supplements and medication, this highlights the need to broaden the content by incorporating physical activity, mental health, sleep and obesity-related risks to offer more comprehensive support.

##### Reliable, practical and meaningful information

A central requirement expressed by participants was that the content provided by the tool must be trustworthy, evidence-based, and aligned with established professional guidelines. Both groups believed that reliable information enhances credibility and helps increase users’ motivation to adopt healthier behaviours. Women voiced their need for a tool that could help them navigate and filter through the abundance of conflicting information found online. As W 10, expressed: “*Google is not your friend; this is precisely a moment for a platform where you can say, ‘This is how it works, and this is how it is done medically.’”* Participants also emphasized that reliable information should explain not just what to do, but why it matters—highlighting the positive impact of certain lifestyle behaviours on both maternal and child health. This kind of framing was seen to make health goals more tangible and meaningful. HCP 9 mentioned*: “Incorporating a few research facts would greatly encourage me…* *For example, the likelihood of a child developing diabetes later in life.”*

In addition to being credible, the content was expected to be concrete and applicable in daily life. Former users of Smarter Pregnancy felt that previous advice had been too generic. They called for more specific and creative suggestions that could be directly implemented. W 2, for example, reflected on her experience: “*Instead of just saying ‘try new vegetables,’ give examples. Parsnip, for instance, is something not everyone eats every day or thinks of…* *Many people, including we, find it hard to fit it all into the evening meal. Maybe also include some snack ideas with vegetables or a vegetable-based lunch. So, offering options instead of just saying ‘eat more vegetables.’”* Participants provided a wide range of suggestions regarding the types of information the tool should offer, which are further detailed in Additional file 5.

##### Tools for interactive tracking, exercise, and goal setting

Besides informational content, participants expressed a desire for interactive features that would allow them to track progress and engage more actively with different lifestyle areas.

In the domain of physical activity, women hoped for access to safe, realistic and professionally approved exercise videos, categorized per trimester and fitness level, either in the program itself or linked to platforms like YouTube. Opinions were mixed about tracking physical activity or movement goals. HCPs and half of the women found this helpful and motivating, especially if it could be shared with healthcare professionals, while the other half considered it unnecessary, particularly if they were already using other tracking apps.

For mental wellbeing, participants requested accessible and varied exercises such as breathing techniques, mindfulness, and short activities for quick stress relief, with links to video or audio resources. Tracking tools, such as mood, sleep, and energy diaries, were also suggested by both women and HCPs to help users reflect and gain insight into their emotional well-being. However, concerns were also raised by HCPs about potential negative effects tracking might have, as HCP 1 explained, “*I get the impression it can be a counterproductive, because it forces them to suddenly reflect on how they’re feeling, and that tends to be quite negative. Reflecting on it could be discouraging.”*

Several other tools were discussed, including accessible and simple recipes to help make healthier choices, a weight journal to increase awareness of trends over time (although some were hesitant about this tool), an integrated smoking cessation program and a medication overview with reminders to help with adequate and timely intake. While some HCPs suggested that gamification elements, like earning points or pregnancy-related rewards, might help boost motivation, most women preferred relying on intrinsic motivation and did not find such features particularly helpful.

##### Personalized, adaptive and goal-driven content

Participants agreed that personalization is crucial for the tool's effectiveness. They wanted the content to be adapted to the user's medical situation, preferences, and personal goals. A customizable interface, allowing users to enable or disable specific modules and tools, was seen as essential to help women focus on what felt relevant and manageable. Setting personal goals was also regarded as a meaningful form of personalization, as it could guide tailored advice both from the tool and from HCPs. Participants did however stress the importance of making these goals stepwise and achievable, to foster a sense of success and reduce the risk of discouragement. As W 10 shared, “*I am a bit heavier. There were things where I wondered: could I even do this if I wasn’t pregnant? That's very demotivating. I think many women face this. When you offer something like this as a standard, you need to make sure it includes things that are achievable and good for you, so that women know: I can trust this.”*

Participants also expressed a need for the tool to be responsive to medical conditions and individual circumstances. For example, W 10 shared her frustration from a previous pregnancy: “*During my first pregnancy, I had severe pelvic instability. If an app then tells me to move more, I just think: ‘screw that.’ I was already happy if I could get up from my chair. These things are just frustrating when they don’t consider the situation.”* Although they acknowledged the technical complexity of such personalization, both women and HCPs believed it was vital to create a supportive and realistic experience.

Moreover, women wished for automatic adaptivity of content based on user input, such as app engagement, survey responses or goals. Former users criticized Smarter Pregnancy for lacking this adaptivity, like receiving fish-based recipes despite dietary restrictions. Adjusting content to fitness levels and pregnancy stages, particularly for physical activity exercises, was also suggested.

Notifications were another area where women wanted personalization options regarding frequency and type, as too many or poorly timed notifications were experienced as intrusive and demotivating by former users. W 3 pointed out the importance of finding balance: “*How do you reach someone without overwhelming them? We already have so much to do, especially as a mother. Growing a child is already challenging. Finding that balance is key”.* Allowing users to adjust the frequency, timing, type, and content of notifications, or disable them entirely, was seen as essential by both HCPs and women for keeping the tool engaging without becoming a burden.

##### Positive, nuanced and informal approach

Participants stressed that the tool should use positive and supportive language, celebrating small achievements and acknowledging efforts rather than focusing on shortcomings. As HCP 2 explained: “*Sometimes, just a simple message like ‘You’re doing great’ can make a big difference. I think being positive and encouraging is really important because they are probably already very good at thinking negatively, like ‘I’m not doing well.’”* HCPs also highlighted the importance of integrating behavioural change techniques, such as psycho-education, distraction strategies, cognitive behavioural therapy, and structured reflection tools to promote sustainable lifestyle change.

Former users perceived the current language of Smarter Pregnancy as judgmental and preachy, assuming unhealthy behaviour and lacking nuance. W 8 noted: “*I already know very well what I am doing right and wrong. It just feels like a confirmation of what I’m not doing well. That doesn’t make anyone very happy.”* Women also expressed frustration with language that seems to oversimplify behaviour change, failing to acknowledge the challenges they face living with overweight. W 9 explained: “*Then you notice that the person writing this has, in my opinion, no clue at all. They don’t understand what it's like to be pregnant or to have overweight or to gain weight. (…) Articles constantly talk about restricting this and that, as if it's all so easy. That's what I mean by stigmatizing language.”* To avoid stigma, women stressed that information should be presented in a nuanced way, recognizing barriers to change and validating the difficulties users may encounter. HCPs also underscored the importance of empathy and acknowledgment in digital health tools.

Additionally, women advocated for an informal and personal tone to create a sense of support and relatability. As W 4 explained: “*I’m not a fan of that preachy and formal language. I just don’t think it's useful. It creates distance. When you download an app, it's because you want help. I think you should get the feeling that someone is standing beside you, supporting and coaching you.”*

Lastly, women mentioned the importance of reassuring messages to reduce anxiety after minor mistakes. W 11 shared: “*You want to do things right. But then you read something you didn’t know—like I did about shrimp. Apparently, you’re not supposed to eat them, but I already had. It would help if the tool included a note saying: ‘If it happens once, it's not the end of the world. It's better to avoid it, but don’t panic.’”*

#### Desired functionalities for a digital lifestyle tool.

##### Streamlined, intuitive mobile application

Participants agreed that a mobile application would be most suitable for a digital lifestyle tool, as it offers accessibility, aligns with modern user expectations, and provides many functionalities. To enhance usability, the app should allow users to log in once and remain signed in. Content should be easily accessible, with a clear structure and user-friendly navigation, where users can effortlessly find and revisit information. Furthermore, participants suggested a reliable but appealing design with vibrant colours.

##### Modular and multimodal design

Participants emphasized the need for a modular structure within the tool and allowing users to enable or disable specific modules. HCP 8 explained what each module should include: “*The app should serve three functions: providing information, offering practical exercises, and enabling self-monitoring. That way, we can structure all modules effectively.”*

Participants favoured a multimodal approach, integrating text, images, videos, and audio formats, such as podcasts, with a combination of in-app content and links to trusted external sources or specialized apps. Additionally, they recommended text-to-speech functionality and explanatory images to enhance clarity, particularly for users with lower language proficiency.

##### Brief surveys with actionable feedback

Both women and HCPs valued short lifestyle surveys to help personalize content and track progress. However, HCPs cautioned that repeatedly answering the same questions without visible improvement could also become discouraging. A clear, easy-to-understand feedback format with a gradual scale and colour indicators was preferred by women and HCPs to show improvements over time. As HCP 1 explained: *“For instance, when you fill in something about your diet, it could be reflected in a score with colors like green, orange, or red, or a similar sliding scale. This way, every time you make small steps, you can see you’re getting closer to the goal.”* Opinions were mixed on peer comparison features (offered by the current program) in this feedback system. While some women found this motivating, others felt it led to self-criticism and unhealthy comparisons. To address this, women suggested an option to enable or disable this feature. Finally, HCPs proposed adding warning signals when results indicate the need for professional support. As HCP 2 noted: “*Certain categories should also serve as an indication for a conversation. So, if something is not going well, you would get an exclamation mark, signalling that this is a good time to initiate a conversation.”*

##### Accessing and sharing data

Women expressed a desire to export and share their information and results outside of the tool. Several options were suggested, including the ability to download, print, or forward data. Former users voiced concerns about losing access to their data once their subscription ended. They highlighted the need to retain this information, particularly if they planned to continue working on their health goals, such as weight loss, after pregnancy. As W 8 explained, “*It would be nice to be able to save your data elsewhere if you want, because if the app stops working, you lose all your data and can’t look back at your pregnancy journey”.* Women also suggested the ability to easily print or forward useful information, such as recipes or shopping lists, to others. W 9 noted, “*It would be great if you could print things or forward them—like a nice recipe you want to share with your mother-in-law, or a shopping list.”*

##### Integrating peer and partner support

Both HCPs and women emphasized the importance of social support in sustaining healthy lifestyle changes. Partner support was seen as helpful. Some women wanted partners to have access to the tool via a shared or parallel version of the tool, while others were reluctant to share access because they wanted the tool to be specifically for themselves. HCPs noted that partners are often less willing to make lifestyle changes. Therefore, both women and HCPs agreed that partner involvement should be encouraged, but made optional.

Peer support, from friends, family, or other pregnant women, was widely seen as beneficial for fostering recognition, motivation and practical tips. Women appreciated learning from shared experiences and often found practical tips from peers more useful and relatable than general health articles. As W 11 illustrated: “*You end up on forums and read experiences from other women. That sense of recognition is nice. It makes you think I’m not alone in this.”* HCPs added that peer support could also reduce stigma, lower barriers for participation and increase motivation. As HCP 2 explained: “*In difficult moments, when you struggle to find motivation yourself, others can sometimes help by saying, ‘Come on, you can do this.’ And that can really pull you through.”*

To facilitate peer support, HCPs and women suggested features like community forums, group chats, or in-person gatherings. However, concerns were raised about the risk of misinformation and potential negative effects on mental well-being by both groups. As W 3 noted: “*The forum is really a pitfall with all the distressing content on there.”* Participants agreed that peer support features should be optional and focus on encouragement rather than medical advice. As W 7 woman explained: “*In some cases, peer advice can be helpful, but you often see—especially in forums—that many people start playing doctor. I believe it should remain accessible.”*

#### Implementation of a digital lifestyle tool.

##### Value and impact of digital tool implementation

Participants identified several benefits of implementing a digital lifestyle tool into obstetric care. HCPs noted that it could offer reliable support between visits, enable more targeted counselling and help manage time constraints during consultations. HCP 13 remarked: “*I only have about ten minutes per patient to address lifestyle changes and obesity risks. The app could help keep counselling concise while still offering in-depth guidance*.” They also saw value in referencing the tool's recommendations during follow-up visits to guide personalized coaching. As HCP 10 explained: “*I’d be curious to see what recommendations the tool provides, so I can refer to them. (…) This way, you can provide targeted coaching based on the advice given by the app.*” Women also valued the idea of having a trustworthy and comprehensive source of information that could offer reassurance and guidance between visits.

Furthermore, both HCPs and women viewed the tool as a potential substitute for brief in-person appointments or calls, offering reassurance whilst preventing unnecessary contact. W 11 noted*: “If my midwife directs me to the app for information in between visits, I wouldn’t have to wait until the next appointment or make small, unnecessary phone calls. It could provide reassurance that I’m on the right track.”*

##### Practical and financial preconditions

Both HCPs and women highlighted the importance of minimal time investment and seamless integration into existing routines as preconditions for successful implementation. HCPs expressed concern that if the tool required too much time, it would either need longer consultations or risk being underused. They suggested embedding the tool into electronic patient records and assigning responsibility for its introduction to professionals with more time, such as nurses or midwives. Women emphasized the need for clear explanations of the tool's purpose and benefits and expressed a strong preference for an all-in-one solution rather than having to use multiple apps. As W 8 noted: “*Right now, I need seven or eight different apps, but if everything was in one place, that would be a real benefit.”*

Opinions on cost varied. Some felt the tool should be free to ensure accessibility, while others believed paying might increase commitment and create a perception of higher quality. All agreed that any costs should be covered by insurance. HCP 6 noted, *“There needs to be a reimbursement system for this, right? For health promotion and education—that would be ideal,”* HCP 8 added: “*This should just be standard care. And standard care should be covered. In the long run, this saves money.*”

##### Facilitating user-provider communication

Furthermore, both groups thought the tool could support communication between users and HCPs.

Participants saw the tool as a complement to, not a replacement for, face-to-face counselling. The ability to share user data with HCPs, either by showing it on-screen, printing it, or syncing it with patient records, was seen as a valuable feature. HCPs believed this could enhance consultation efficiency, provide valuable insights, help tailor advice more effectively and improve continuity. Both groups valued the ability to structure conversations around concrete data points. As W 6 stated: “*If I could print out my progress and bring it to my midwife, it would make conversations easier. You’d have concrete data to show whether you’re improving or struggling.*” Additionally, they believed this would lead to a reduction in repetitive conversations and improvement of mutual understanding between user and HCP. As W 5 noted, “*It would be helpful to show what I am already doing, rather than having to explain everything repeatedly. If I can share it once, it's much easier, and I won’t forget anything, like what I ate last week.”* And HCP 8 explained: “*Perhaps the tool could help in the HCP-patient relationship, showing that the woman is trying, that she's moving, which might lead to more understanding from the HCP. This could also motivate HCPs to better support these women.”*

Another proposed feature was a messaging function between users and HCPs to lower the threshold for asking questions and potentially reduce the need for short in-person visits, although HCPs voiced concerns whether they would have enough time to manage such communication. As an alternative, some HCPs suggested adding alerts to flag when users should seek professional guidance, to encourage timely discussions about emerging concerns.

Despite the perceived benefits of data sharing, some women preferred to keep their data private, fearing constant monitoring. Others warned that it would be demotivating if shared information went unacknowledged. W 1 remarked*: “If there isn’t a clear system in place, it can be discouraging to log everything into the app, thinking my HCP is seeing it, only to find out they’re not even looking at it. Then, in a consultation, they just tell me what to do without even checking what I’ve already done.”* To address this, participants stressed that data-sharing should be user-controlled, with clear expectations about whether and when HCPs would review the input. Additionally, shared data should be linked to follow-up conversations to ensure it is acknowledged and used meaningfully.

##### Effective rollout and sustained use

To support long-term engagement, women emphasized that the tool should be framed as a supportive resource to help improve personal health, rather than as a directive or monitoring device. HCPs proposed offering the tool through channels like welcome emails, brochures, or provider websites, and suggested that it should be available to all patients—not just those with obesity—to promote consistent implementation. Both groups also advocated for making the tool available in multiple languages to enhance inclusivity.

Although the tool was seen as especially beneficial for women with limited health knowledge, HCPs questioned whether it would reach those most in need, particularly if motivation to engage was low. Early introduction, preferably in early pregnancy or even during the preconception period, was considered most effective. HCPs also pointed to the importance of continued support beyond pregnancy, especially in preparation for future pregnancies. They noted that existing apps typically overlook postpartum care and interpregnancy health, while a tool that bridges this gap could offer long-term value. Women echoed this need for continued support, explaining that lifestyle guidance is also needed in the postpartum period. As W 6 shared: *“It would be disappointing if the app stopped working once the baby is born. I think it's crucial to keep the support going after pregnancy. After pregnancy, this is even more important because the extra kilos are there and need to come off.”*

## Discussion

### Principle findings

This study explored (a) the current perspectives and practices related to healthy lifestyle behaviours and lifestyle care for pregnant women with obesity, and (b) the needs and preferences of both women and HCPs for the development and implementation of a digital lifestyle intervention.

Regarding the first aim, women described healthy living as encompassing nutrition, physical activity, mental well-being, and rest, but encountered numerous barriers, including physical discomforts (linked to both pregnancy and obesity), emotional stress, limited knowledge, financial constraints, and fear of judgment. Both women and HCPs identified the child's health as a primary motivator for change, and valued goal setting and more knowledge about healthy lifestyle and risks of obesity as supportive strategies. Nevertheless, lifestyle care varied widely in practice. Women often felt unsupported, receiving generic or overly medicalized advice that did not account for the challenges associated with obesity or their physical health. HCPs reported time pressures, unclear roles, limited confidence of expertise, and concerns about stigma as barriers to offering consistent, tailored guidance. These factors contributed to missed opportunities for effective, personalized support.

Regarding the second aim, both women and HCPs endorsed the development of a user-friendly, comprehensive digital lifestyle tool addressing core lifestyle domains, including nutrition, physical activity, mental well-being, sleep, and substance use. HCPs additionally requested including content on supplements, medication use, and obesity related pregnancy risks. Across groups, participants prioritized reliable, evidence-based, and practical information presented in accessible formats, explaining both the relevance of lifestyle changes and how to implement them in daily life.

Participants envisioned a modular, intuitive mobile app that combines educational content with self-monitoring features such as activity and mood tracking, exercise videos, and goal setting. However, HCPs cautioned that such features should be carefully contextualized to avoid negative emotions and both groups emphasized the importance of adaptation for women with obesity. For example, exercise videos should reflect varying levels of mobility, mood tracking should avoid inducing negative emotions or guilt, and goal-setting tools should support small, achievable steps tailored to individual capacity. Peer and partner support were considered beneficial but should remain optional due to concerns of misinformation and varying personal preferences. Personalization emerged as a central design requirement, with content, notifications and tracking features tailored to users’ medical status, pregnancy stage, preferences, and personal goals. Customizability of the interface was also deemed important to enhance relevance. Both groups stressed the importance of a supportive and non-stigmatizing tone. HCPs additionally recommended embedding behaviour change techniques to support sustainable change.

Regarding implementation, both groups recognized the substantial value of integrating a digital lifestyle tool into routine obstetric care. HCPs highlighted benefits such as improved continuity between visits, enhanced counselling efficiency and reduced time pressure during consultations. Women valued reliable and accessible information to support them during and between visits. Successful implementation was seen to depend on minimal time investment, integration into existing systems, clear communication about the tool's purpose, user control over data sharing, and affordability. Sustained engagement was linked to early introduction, ideally starting before or at the beginning of pregnancy, and continued availability throughout pregnancy and the postpartum period.

### Comparison with literature

Our findings align with previous studies that identify pregnancy as both a motivator and a barrier to lifestyle change. As in earlier research, women in our study cited concern for foetal health and increased awareness as key motivators.^36–38^ Consistent with prior work, participants also reported barriers such as physical and emotional discomfort, limited knowledge, stigma, and lack of tailored information.^12^^36–38^ However, unlike other studies, our participants did not report major concerns about practical access, cultural barriers, inconsistent advice, or the perception of pregnancy as a justification for unhealthy behaviours.^12,27^^36–38^ Additionally, our study highlights goal setting as a distinct motivator, which has received limited attention in the existing literature.

Our findings confirm and extend earlier work on desirable features of digital lifestyle tools for pregnancy. Like others, our participants emphasized the need for evidence-based, practical content focused on nutrition, physical activity, and weight management.^27,30,31^ However, our participants also called for broader coverage of less commonly addressed topics such as mental health, sleep, substance use, and obesity-related pregnancy risks. While the preferred format, a mobile app combining education with self-monitoring and goal-setting, was consistent with prior research,^24,31^ our HCPs uniquely stressed the importance of contextualizing tracking features to avoid adverse emotional responses, a concern previously only noted by women.^30^

Furthermore, although prior studies described peer and partner support as helpful,^24,31,39^ our participants clearly preferred making these features optional due to potential disadvantages for example misinformation. The emphasis on personalization in our findings supports earlier calls to tailor content to pregnancy stage, health status, and user preferences.^24,27,39^ Additionally, our participants highlighted the importance of supportive, non-judgmental language, an aspect not described in prior work.

Finally, while Willcox et al. reported a mismatch between women and HCPs, with women viewing digital tools as a valuable addition to care, but HCPs perceiving them as parallel to routine practice with limited added value,^24^ our findings showed alignment between both groups. In our study, HCPs explicitly recognized the potential of digital lifestyle tools to improve continuity of care, streamline counselling, and reduce time pressure during consultations. Both women and HCPs emphasized that digital lifestyle tools should be fully embedded within routine obstetric care. Furthermore, whereas prior studies gave limited attention to practical implementation, our study combines user-centred design with specific implementation needs. This offers a more comprehensive foundation to guide the real-world development and adoption of digital tools for women with obesity in obstetric care.

### Strengths and limitations

To our knowledge, this is the first study to examine both pregnant women with obesity and HCPs to identify shared and diverging needs regarding lifestyle support and the development of digital lifestyle interventions. This dual-perspective approach is a key strength, as it supports the design of interventions that are both user-centred and feasible to implement in clinical practice.

However, several limitations should be noted. Most participants were Dutch, which may limit applicability to other cultural contexts. While we aimed for cultural diversity and included participants with varying backgrounds, generalizability beyond the Dutch healthcare setting remains uncertain. However, our findings are consistent with studies conducted in other countries, suggesting a degree of transferability. Despite using purposive sampling, included participants may have been more motivated or digitally literate than average, potentially introducing selection bias.

Additionally, the qualitative nature of the study limits generalizability. Social desirability bias may have influenced participant responses, particularly in group settings. The use of example content during focus groups could have steered discussions; however, this strategy helped elicit richer insights into user needs. Participants also received a modest financial reimbursement, which may have discouraged some from expressing strong criticism, although we believe this influence was limited. Finally, moderation by multiple researchers may have introduced some variation in facilitation, but also helped reduce individual interviewer bias.

## Conclusions

This study highlights the need for a personalized, user-friendly digital lifestyle tool that delivers practical, evidence-based information and integrates seamlessly into obstetric care. Importantly, participants emphasized the need for non-judgmental communication, flexibility, and adaptability to individual needs and preferences.

By combining user-centred design insights with practical implementation considerations, this study provides a foundation for developing digital lifestyle tools that are both acceptable to users and feasible for integration in real-world obstetric settings. These findings offer concrete guidance for the development of more effective and clinically relevant interventions that can enhance continuity of care, improve engagement, and support meaningful behaviour change. Building on these insights, we will proceed with the development of *Smarter Pregnancy Plus*, a tailored digital intervention for women with obesity, which will be evaluated in a randomized controlled trial to assess its effectiveness and impact on maternal pregnancy and birth outcomes.

## Supplemental Material

sj-docx-1-dhj-10.1177_20552076251408518 - Supplemental material for Towards effective digital lifestyle interventions for pregnant women with obesity: A qualitative study exploring women's and healthcare providers’ perspectives Supplemental material, sj-docx-1-dhj-10.1177_20552076251408518 for Towards effective digital lifestyle interventions for pregnant women with obesity: A qualitative study exploring women's and healthcare providers’ perspectives by Rianne J de Bruin, Caroline A Figueroa, Pam ten Broeke, Kim N de Jonge, Melek Rousian, Régine PM Steegers-Theunissen and Ageeth N Rosman in DIGITAL HEALTH

sj-pdf-2-dhj-10.1177_20552076251408518 - Supplemental material for Towards effective digital lifestyle interventions for pregnant women with obesity: A qualitative study exploring women's and healthcare providers’ perspectives Supplemental material, sj-pdf-2-dhj-10.1177_20552076251408518 for Towards effective digital lifestyle interventions for pregnant women with obesity: A qualitative study exploring women's and healthcare providers’ perspectives by Rianne J de Bruin, Caroline A Figueroa, Pam ten Broeke, Kim N de Jonge, Melek Rousian, Régine PM Steegers-Theunissen and Ageeth N Rosman in DIGITAL HEALTH

## List of abbreviations

BMI: Body mass index
COREQ: Consolidated criteria for reporting qualitative research
HCP: Healthcare provider
SRQR: Standards for reporting qualitative research
W: Woman
