# Supplementary material for: Towards effective digital lifestyle interventions for pregnant women with obesity: A qualitative study exploring women's and healthcare providers’ perspectives
Source: Digit Health. 2026 Mar 23;12:20552076251408518. doi: 10.1177/20552076251408518 (PMC13009833; doi:10.1177/20552076251408518)
Supplement: sj-docx-1-dhj-10.1177_20552076251408518 - Supplemental material for Towards effective digital lifestyle interventions for pregnant women with obesity: A qualitative study exploring women's and healthcare providers’ perspectives [file sj-docx-1-dhj-10.1177_20552076251408518.docx]

## Additional file 1 – Screenshots from the existing Smarter Pregnancy program


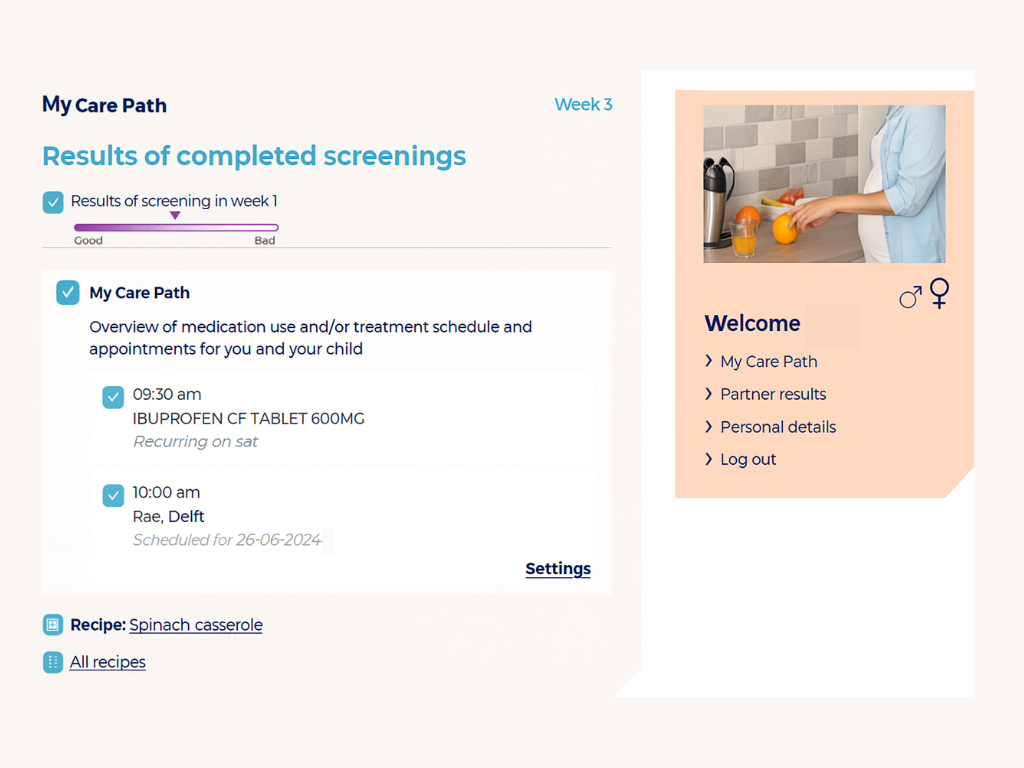


**Figure 2**: Screenshot showing the user’s homepage after logging in to the program.

_On the left, from top to bottom: summary of the results from week 1, the registered medication and appointments and the last recipe this user received._

_On the right: the menu, in which a user can direct to her own care pathway, her partner’s results, her personal details or sign out._


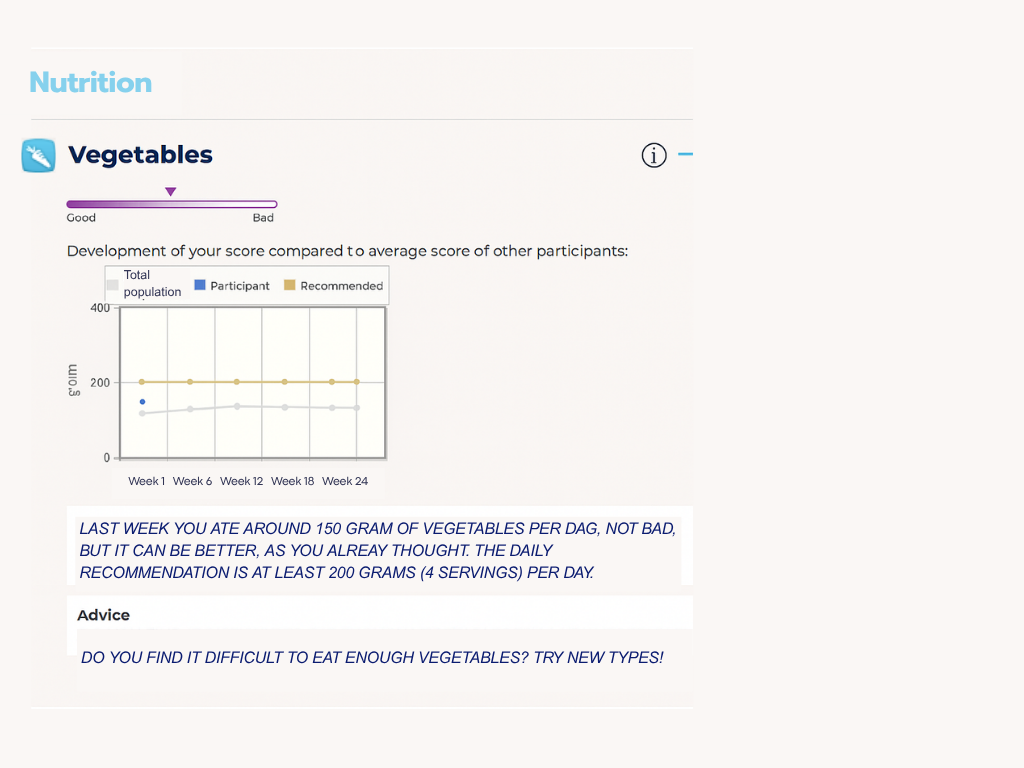


**Figure 3**: Screenshot showing results for vegetable intake.

_The participant’s score (blue) is shown compared to the total population (grey) and the recommended amount (yellow). As the program progresses, the user can see her own progress, which will then be depicted as a blue line in the graph._

_Beneath the graph, it shows the feedback and advice this user received._


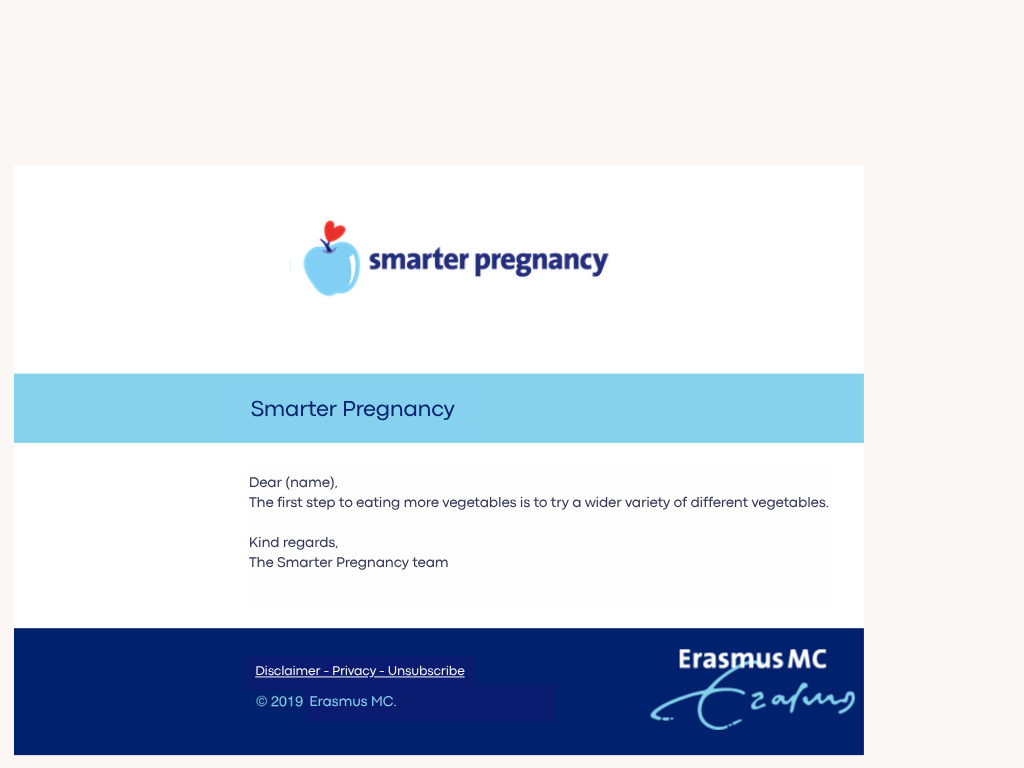


**Figure 4**: Screenshot of advice a user received by email.

## Additional file 2: Suggestions of content provided to focus groups participants

Activity weekly overview
Bonus point system
Breathing exercises
Check-in for energy levels
Chatbot
Daily wellness tips
Diary
Exercise program at level
Exercise suggestions
Exercise videos
Goal registration
Group support
Language settings
Live streaming training sessions
Medication overview
Mindfulness exercises or meditation
Mood diary
Movement tracking with the use of a smartwatch
Nutritional advice
Overview sharing with healthcare provider
Partner support
Pedometer
Personal training plan
Personalized profile
Pregnancy test registration
Psychoeducation
Quit smoking program
Recipes
Registration of behaviour change agreements
Reminder to get moving (nudges)
Sleep diary
Virtual mental health coach
Weight diary

Additional file 3: Semi-structured interview guides pregnant women and former users

[INTRODUCTION]

Today, I would like to talk to you about health, pregnancy, and digital lifestyle tools that may support you during this period. We want to learn about your experiences as someone who is currently pregnant or a former user of the Smarter Pregnancy program. Your input will help us improve and develop a new lifestyle tool for pregnant women with obesity.

This interview is not a test—there are no right or wrong answers. Your experiences and wishes are very valuable, and your name will not be used. No one will know you participated. The interview will take about thirty minutes.

With your permission, I would like to record the conversation so we can later convert it into text for analysis. I may also take notes while you speak. If you have any questions during the interview, please feel free to ask. If there are questions you prefer not to answer, you can skip them without explanation.

[FORMER USERS]

- What did you think of Smarter Pregnancy?
- What did you like?
- What did you not like? What would you change or what did you miss?
- What did you think of the tips, facts, and recipe messages you received (content, frequency)?
- Did it fit your needs?

In the new version of Smarter Pregnancy, we plan to include new features and improved messaging.

- What do you think of this?
- Is there a component you find particularly important—or not at all?

[STRESS AND MENTAL HEALTH]

- What causes or caused you stress during pregnancy?
- What helps you reduce stress during pregnancy?
- Are there things you would like to do differently in dealing with stress? What do you need *(from yourself, others, work, etc.)*?
- Could an app/website help you with this? Why or why not?
  - Are there apps/websites you use or would like to try?
  - What do you like about these apps/websites?
  - What do you not like?
- How could an app/website support your mental well-being activities?
  - What type of exercises could help you?
  - Would it be enough for the app/website to tell you where to find helpful exercises?
  - What types of messages would help you?

[PHYSICAL ACTIVITY]

Physical activity is an important part of a healthy lifestyle. This includes not only sports, but also walking, dancing, cycling, and household activities.

- What kind of physical activity do you do in a week? Has this changed because of pregnancy?
  - What caused the change? *(Fatigue, nausea, fear of harming the pregnancy, etc.)*
- What activities would you like to do more often?
  - What do you enjoy? What do you dislike?
  - What helps you stay active, and what does not?
- What would you like to see in an app/website regarding physical activity? *(Exercises, information, videos, etc.)*
  - What appeals to you?
  - What does not appeal to you?
- Would it be enough if the app/website only provided information on where to find exercises, or should it offer exercises directly?
- Where do you find information about physical activity during pregnancy?
  - How easy is it to find this information?
- Do you set goals for physical activity? What are they?
  - Would setting goals help you?
  - What would help you achieve these goals?
  - How does it affect you if you reach—or do not reach—a goal?
- If you could design the movement section of the app/website yourself, what would it look like?

[ADDITIONAL HELP]

- Would you want a (video)consult with someone trained in lifestyle coaching?
  - Would you prefer this at the beginning or later?
  - Once or more often?
  - Or would you prefer to ask your midwife?

[FORM AND MESSAGING]

- Should Smarter Pregnancy be an app or a web application?
- How should it address you?
  - What type of messages would help?
  - How many messages would you want?
  - When?
  - Would you want to choose the topics?
- If you received a fitness tracker, would you use it?
  - Do you already have one? Why or why not?
  - Would you wear it consistently?
  - Could this help you be more active?
- How do you feel about having a community of other pregnant women to exercise together, share messages, or motivate each other?
  - What do you like or dislike about this idea?
  - What would this look like for you? *(Examples: forum, one-on-one buddy, walking group).*

[ACCESSIBILITY]

- How can we make the app/website clear and easy to use for you?
- What type of information/visuals do you prefer in an app/website? *(Text, images, videos, tables, graphs, etc.)*
- Many people find apps and account creation difficult. How is that for you?
  - If difficult: Do you have someone who can help?
- How can we make sure the app/website feels like it is meant for you?
- Should the app/website be available in other languages?
- Would spoken text be helpful?
- Would you be willing to pay for such an app/website?
  - How much? What would this depend on?
- What information are you comfortable entering in an app/website (*medical info, personal info, weight, mood, etc.)*?
- Would you be comfortable sharing app/website data with a healthcare provider?
  - For how long may they access your data?
  - How would you like to manage or delete your data?
- What would help you continue using the app/website over time?
- How should the app/website function to motivate continued use?

[GENERAL QUESTIONS]

- Did you discuss lifestyle with your midwife or doctor early in pregnancy?
- What did you think of that conversation?
- Could an app/website replace this discussion?
- How should an app/website be recommended to you? How should a provider introduce it so that you feel interested?

Additional file 4: Semi-structured interview guide healthcare professionals

[INTRODUCTION]

Today, I would like to talk to you about your perceptions regarding a digital lifestyle program for pregnant women with obesity. Your input will help us improve and develop a new lifestyle tool for pregnant women.

This interview is not a test—there are no right or wrong answers. Your experiences and wishes are very valuable, and your name will not be used. No one will know you participated. The interview will take about thirty minutes.

With your permission, I would like to record the conversation so we can later convert it into text for analysis. I may also take notes while you speak. If you have any questions during the interview, please feel free to ask. If there are questions you prefer not to answer, you can skip them without explanation.

[SMARTER PREGNANCY]

- Do you know Smarter Pregnancy?
  - **YES:** Do you use Smarter Pregnancy in your practice?
  - **NO:** Smarter Pregnancy is a digital lifestyle coaching program that helps couples trying to conceive—and pregnant women—improve key lifestyle behaviors. After completing a short screening covering aspects such as nutrition, folic acid use, substance use, and physical activity, the program generates an overview of behaviors that may need improvement. Participants then begin a 26-week tailored coaching program. During this period, they receive education, tips, motivational messages, and rewards—such as vouchers for pregnancy tests or folic acid supplements—as well as seasonal recipes aimed at supporting healthier lifestyle habits.
- **I will show you some examples (figures 2, 3, 4 are shown).**
  - What are your thoughts on Smarter Pregnancy?
  - What works well?
  - What could be improved?

[DEVELOPMENT NEW TOOL]

As you know, we are working on improving the program and will evaluate the effect of Smarter Pregnancy+ in a randomized study. To achieve this, it is important for us to understand what healthcare providers consider essential for the new version to function successfully in practice.

- What would you like to see included in Smarter Pregnancy+?
- Which components, functionalities, or other requirements are important to you?
- How could Smarter Pregnancy+ support your work?
- How could it be integrated into your current workflow?
- What would you want to gain from using it?
- How would you ideally use the tool?
- What would you personally add to the program?
- Which functionalities do you consider necessary?
- Would you want to be able to view patient data?
- What added value would such a tool need to offer for you to use it in daily practice?
- What would be required to make this tool practical for use in your everyday work? How would you prefer to use it?

[IMPLEMENTATION]

We aim to offer Smarter Pregnancy+ to every pregnant woman with obesity.

- In your view, what requirements must the tool meet to make this feasible?
- What is needed to ensure that you would actually use it?
- When is the tool user-friendly for you?
- Would you want access to patient-entered data?

## Additional file 5: Suggestions for information to be included in the digital lifestyle intervention

| **Nutrition and supplements** | |
| --- | --- |
| Encourage eating vegetables throughout the day. | Suggest fun ways to prepare and enjoy healthy meals. |
| Educate about proper portion sizes. | Offer tips for bringing nutritious food to work. |
| Recommend healthy ready-made options from supermarkets. | Share ideas for balanced snacks and lunches. |
| Advise on healthy ready meals. | Offer budget-friendly ways to eat healthily. |
| Clarify what to eat and avoid during pregnancy. | Tailor dietary advice to each stage of pregnancy. |
| Help manage cravings during pregnancy. | Provide guidance for a healthy vegetarian diet. |
| Emphasize benefits of reducing simple carbs. | Provide a nutrition module to centralize dietary advice. |
| Ensure information aligns with official dietary guidelines. | Explain daily meal timing and structure. |
| Highlight the importance of supplements, especially folic acid. |  |
| **Exercise and activity** | |
| Motivate with fun and engaging ways to exercise. | Emphasize movement in daily surroundings (not just gyms). |
| Suggest alternatives to running and gym workouts. | Share tips for being active at home. |
| Acknowledge housework as a form of physical activity. | Educate about safe exercise options during pregnancy. |
| Clarify safe heart rate limits during physical activity. | Promote local sports or activity programs. |
| Inform about discounted sports opportunities. | Explain bodily sensations related to movement. |
| Help identify warning signs or limits during exercise. | Summarize the benefits of physical activity. |
| **Mental health** | |
| Encourage regular rest and downtime. | Provide practical relaxation strategies. |
| Promote “me time” and personal care moments. | Share actionable stress reduction strategies. |
| Normalize stress and emotional fluctuations. | Explain how maternal stress can impact the baby. |
| Educate about hormone-driven emotional changes. | Clarify common emotions per trimester. |
| Offer guidance to improve sleep. | Highlight mental wellbeing as core to prenatal care. |
| Reframe stress as a potential for personal growth. | Offer CBT techniques like the G-schema. |
| Provide psychoeducation about feelings and behavior. | Include FAQs and relatable peer experiences. |
| **Behavior change** | |
| Suggest achievable healthy actions. | Provide alternatives and distractions for unhealthy habits. |
| Use structured tools like CBT or G-schema to support change. | Help women prepare for setbacks through a relapse prevention plan. |
| Clarify why advice and behaviour change matter. | Educate on what healthy choices look like. |
| Link behaviour changes to health of mother and baby. | Reinforce motivation by linking lifestyle to mother and baby's health. |
| **Pregnancy knowledge** | |
| Provide tips for discussing needs with employer. | Advise on reducing activity before bedtime. |
| Offer information on foetal development. | Explain common physical experiences during pregnancy. |
| Identify and explain common pregnancy complaints. | Offer guidance on 22-week vaccine, childcare, and parenting. |
| Provide a checklist for baby-related preparations. | Share information on postpartum care (feeding, sleep). |
| Offer advice for handling pregnancy symptoms like nausea. | Clarify physical boundaries for movement during pregnancy. |
| Explain how symptoms can be educational or meaningful. | Share financial information about childcare subsidies. |
| Describe physical and emotional changes during pregnancy. | Educate on hormonal effects and emotional wellbeing. |
| Inform about the importance and benefits of healthy behaviour. | Highlight pregnancy-related changes in a reassuring way. |
| Focus messages on both mother and child. | Share risks of overweight and how to prepare. |
